# A New Description of the Entire Body (in Chinese)

**Published:** 1858-01

**Authors:** 


ART. V.—*Ts'ün T'ai San Lun.* "*A new Description of the entire Body.*" By BENJ. HOBSON, M.B. Published at the "Wai Oi" Hospital, Canton, China.

THE book whose uncouth title heads this article is an octavo volume of 152 pages, printed in the Chinese character. It is an epitome of the principal facts of anatomy and physiology as they are known to the profession in Europe and America. Beginning at osteology, the various structures of the system and the functions of the organs are described, and practical remarks on medicine and surgery are introduced. The treatise concludes with an account of the development of the fœtus in utero, and some of the accidents of parturition. It is illustrated with numerous woodcuts—copies of those in common use in modern works on anatomy and physiology.

It is not easy to appreciate the difficulty of transferring a science into a new language where a nomenclature has to be invented and words applied to the description of objects hitherto unknown. This was the labor undertaken by Dr. Hobson, for his work is the first attempt to introduce to the inhabitants of the celestial empire a true account of the structure and functions of the human system. That he has succeeded in his effort to a very encouraging extent is shown by the estimation in which his book is held by intelligent natives. A large edition has been published by a wealthy native merchant, with an introduction acknowledging its superiority, yet taking exceptions to some parts as altogether chimerical.

The father of Yeh, the present Governor-General of Canton, published a very fine edition of the plates. They make eight sheets, a foot wide by three long, and are a complete set of anatomical plates. A higher compliment could not be paid to any book, especially one of foreign origin, for it must be remembered that educated natives look with profound contempt upon anything which comes from the "barbarian or red-haired devils."

The benefit which Dr. Hobson has attempted to confer upon 300,000,000 of the human family can only be estimated by considering that they know nothing of anatomy and physiology, and that not even an attempt is made at the practice of surgery. Space does not permit us to enlarge upon the general state of the profession and the practice of medicine among our antipodes. Of this more anon.

